# Non-invasive biomarkers to diagnose and monitor eosinophilic esophagitis: a systematic review

**DOI:** 10.3389/fmed.2025.1607306

**Published:** 2025-06-26

**Authors:** Sophia-Louise Noble, Richard Tyrrell, Thomas C. Mules, Stephen Inns

**Affiliations:** ^1^Malaghan Institute of Medical Research, Wellington, New Zealand; ^2^Department of Medicine, University of Otago, Wellington, New Zealand; ^3^Hutt Hospital, Lower Hutt, New Zealand

**Keywords:** eosinophilic esophagitis, non-invasive biomarkers, biomarkers, diagnosis, disease monitoring

## Abstract

**Background and aims:**

Current assessments for diagnosing and monitoring eosinophilic esophagitis (EoE) are invasive, time-intensive and costly. The development of non-invasive biomarkers that are sensitive and specific for EoE is paramount. We aimed to provide a comprehensive update on the latest biomarker discovery research in EoE and discuss the current state of the field.

**Methods:**

PubMed, Web of Science, Medline and Embase were searched for studies on non-invasive biomarkers for EoE. Extracted studies were analyzed for risk of bias and relevant data was extracted, including study design, participants, information on controls, biomarker detection method, biomarkers studied, and biomarkers for which statistical significance was found.

**Results:**

Of the 239 studies identified, 37 articles met the inclusion criteria and were included in the systematic review. Across these studies, over 80 biomarkers were evaluated as potential non-invasive tools for diagnosing and monitoring EoE. While the most commonly investigated biomarker was peripheral eosinophil count (PEC), overall PEC demonstrated limited reliability. Other emerging biomarkers, including eosinophil-derived proteins (e.g., EDN, MBP-1) and cytokines (e.g., eotaxin-3), showed promise, although findings were inconsistent between studies. Esophageal-specific sampling methods, such as the Cytosponge, esophageal string test (EST), and esophageal brushings, demonstrated strong correlations with histologic eosinophil counts and disease activity, particularly through the measurement of eosinophil-associated proteins.

**Conclusion:**

Esophageal-specific sampling methods show significant promise for accurately diagnosing and monitoring EoE, particularly through eosinophil-derived biomarkers, such as eosinophil-derived neurotoxin (EDN). Furthermore, these methods were better tolerated and more cost-effective compared to endoscopy and biopsy.

## Introduction

Eosinophilic esophagitis (EoE) is a chronic immune-mediated disease of the esophagus, characterized by eosinophil-predominant inflammation in the esophagus and symptoms of esophageal dysfunction, such as dysphagia and food impaction (1). The incidence and prevalence of EoE is currently on the rise. Prevalence is higher in males than females, and higher in people of European ethnicity than Asians and African Americans (2). The etiology of EoE is complex and multifactorial, involving genetic risk factors, environmental exposures and allergen-mediated type 2 inflammation, and remains incompletely understood (3).

Currently, diagnosing EoE and monitoring for disease progression and treatment responsiveness requires esophageal biopsies and histopathological assessment of esophageal eosinophils (4). This requires repeat upper gastrointestinal (GI) endoscopies, which are invasive, time-intensive and costly. Moreover, there is marked variability of eosinophil infiltration along the esophagus, resulting in inconsistencies in diagnoses (4). Therefore, there is a paramount need for less invasive diagnostic and monitoring methods that are sensitive and specific for EoE. Some of these tests do involve instrumentation of the oesophagus in some form, however all avoid endoscopy. While the argument could therefore be made that the tests are “minimally-invasive” rather than “non-invasive” we have used the nomenclature found commonly in the literature of “non-invasive” to refer to any test that does not use endoscopy in its application. In recent years, various studies have been conducted to address this need, however, to date, non-invasive biomarkers have not been adopted into clinical practice, with endoscopy with biopsy remaining the gold standard for diagnosing and monitoring EoE.

Hines et al. previously published a systematic review of the topic, summarizing the literature up until June 6, 2017 (5). Here, we present an in-depth and up-to-date systematic review of studies that have been conducted since that date which have investigated non-invasive biomarkers for EoE. This review summarizes the current state of biomarker research in EoE and discusses emerging non-invasive biomarkers categorized by their collection source: peripheral blood, oral cavity and saliva, exhaled breath, urine, and non-invasive esophageal sampling.

## Methods

### Eligibility criteria and literature search

This systematic review was conducted and reported according to the Preferred Reporting Items for Systematic Reviews and Meta-analysis (PRISMA) guidelines and statement (6). Articles were required to be primary studies (not reviews) based on human research and were included based on diagnostic criteria for EoE. In addition, articles were required to study a non-invasive biomarker, defined as a biomarker that did not require an endoscopy. On July 19th 2024 the first author systematically reviewed English-language articles using PubMed, Web of Science, Medline and Embase. For this systematic review studies dating from 1 July 2017 to 19 July 2024 were included in the research. The following search terms were used: eosinophil*, hypereosinophil*, esophagus*, serologic* marker*, marker*, biomarker*, Cytosponge, brush, string test, non-invasive, minimally invasive, semi-invasive, non-endoscop*. The full search strategy is reported in Supplementary material 1. The initial search yielded 239 results (PubMed: 36, Web of Science: 44, Medline: 41, Embase: 118).

### Study selection

Articles that fulfilled the above inclusion criteria and contained all search elements were retrieved and entered into the Rayyan application. Rayyan is an online tool for systematic reviews which facilities blind article screening (7). Duplicates were removed, resulting in 127 articles. The first author and second author worked independently to establish whether each of the identified abstracts met the eligibility criteria. Inclusion was determined using the population, intervention, comparator, outcome (PICO) criteria: the study must include patients with EoE (population), the use of a non-invasive biomarker (intervention), with a comparison to standard invasive methods or no biomarker (comparator), and include analysis of patient outcomes, such as diagnostic accuracy, ease of use etc. (outcome). If reviewers were uncertain or disagreed on whether to include or exclude a study, disagreements were resolved by consensus with a third reviewer.

The full-text publications were reviewed after abstract screening to determine inclusion or exclusion. If reviewers were uncertain or disagreed on whether to include or exclude a study, disagreements were resolved by consensus with a third reviewer.

### Assessment of methodological quality

To assess risk of bias, the first and second author independently assessed the methodological quality of each study during the review of the full-text publications, using the *Cochrane Handbook for Systematic Reviews of Diagnostic Test Accuracy* risk of bias tool; the revised Quality Assessment of Diagnostic Accuracy Studies (QUADAS-2) (8). Using the QUADAS-2 tool four key domains were evaluated: patient representation, index test, reference standard, and flow and timing. Each domain was scored as low, high, or unclear, according to the QUADAS-2 assessment criteria.

### Data collection

Relevant data was extracted into a Microsoft Office Excel spreadsheet by the first author, and these data were re-checked by the second author. Any disagreements were resolved by author consensus. The data spreadsheet included the following: first author, year of publication, age of participants, study design, sample size, information on study controls (e.g., healthy, atopic, inactive disease), biomarker detection method, a complete list of all biomarkers studied, and a list of all biomarkers for which statistical significance was found.

## Results

**Figure 1:**
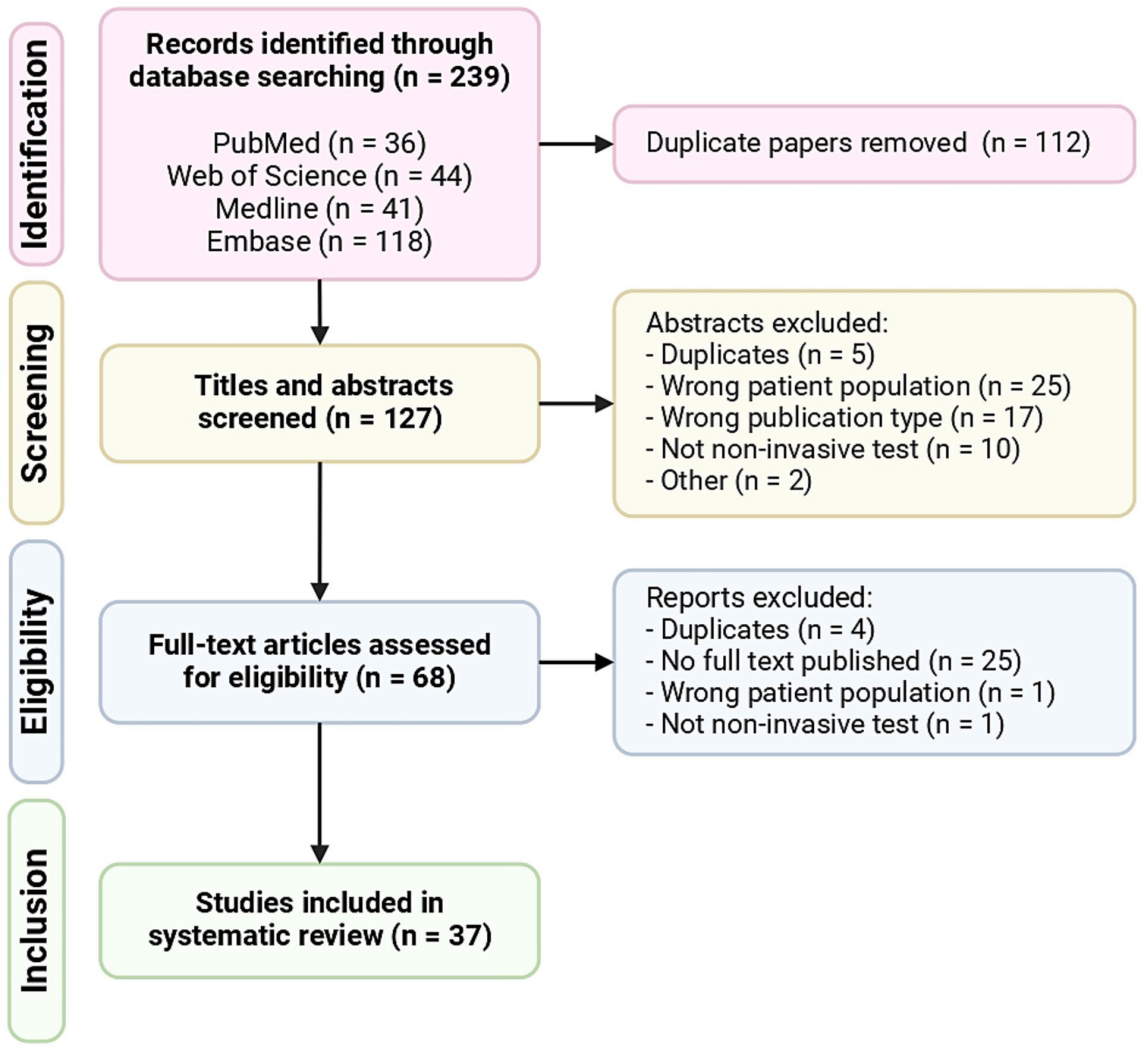
PRISMA (Preferred Reporting Items for Systematic Reviews and Meta-Analyses) flowchart of included articles.

**Figure 2:**
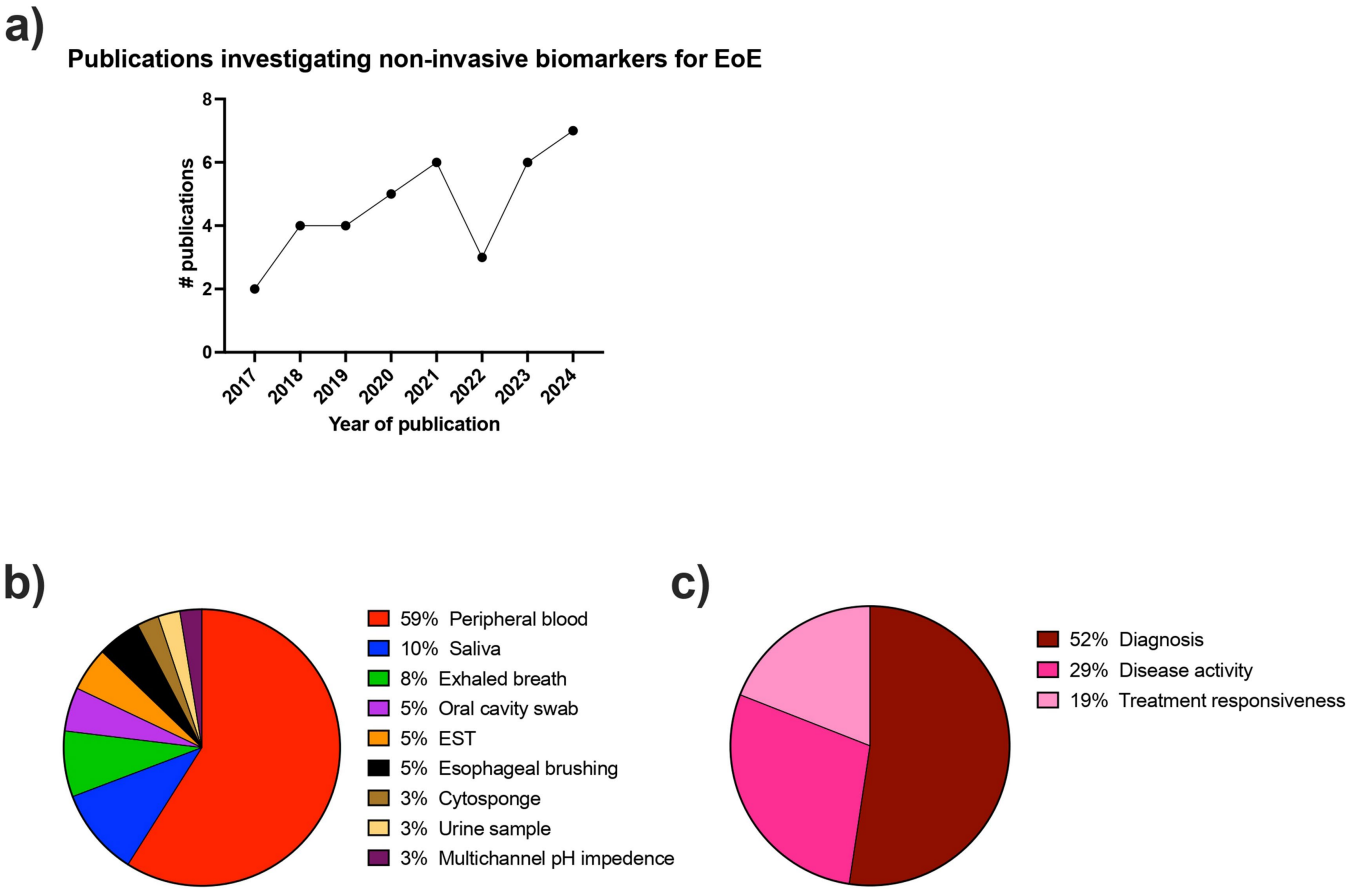
Descriptive characteristics of included articles. **(a)** Number of publications per year (year defined as July to July) investigating non-invasive biomarkers for the diagnosis and monitoring of EoE. **(b)** Proportion of collection methods used in the included articles for non-invasive biomarkers for EoE. **(c)** Proportion of applications used in the included articles for non-invasive biomarkers for EoE.

The initial literature search resulted in 239 results. Following abstract review and full-text review, 37 articles met all of the inclusion criteria and were included in the systematic review (Figure 1). The descriptive characteristics of the included articles are highlighted in Figure 2. The details of all 37 studies investigated in the review are presented in Supplementary Table 3.

### Descriptive characteristics of included studies

The number of studies investigating non-invasive biomarkers for EoE has been steadily increasing over time since 2017 (Figure 2A). There was a large breadth of biomarkers investigated, with over 80 different biomarkers evaluated for the diagnosis and monitoring of EoE. In the included studies, the sample collection and detection methods varied widely and included peripheral blood, saliva, exhaled breath, oral swabs, esophageal string test (EST), esophageal brushing, Cytosponge, urine, and multichannel pH impedance. Figure 2B shows the frequency of each of the detection methods. The most commonly investigated biomarker source was peripheral blood.

Twenty-six studies included non-EoE controls, six studies compared patients with inactive and active EoE, three studies included patients with gastroesophageal reflux disease (GERD), and only one study included atopic participants as controls. Regarding the intended biomarker application, 52% of studies were associated with EoE diagnosis, 29% with monitoring disease activity, and 19% with monitoring treatment responsiveness (Figure 2C).

### Methodological quality assessment

Quality assessment was performed using the QUADS-2 tool as described in the methods section above. Details of the methodological quality assessment are presented in Supplementary Table 2 and Supplementary Figures 1A,B. Overall, risk of bias was variable across studies. While some studies demonstrated low risk across all four domains, many showed high or unclear risk—particularly in the domains of patient selection and index test, often due to non-consecutive recruitment, lack of pre-specified thresholds, or inadequate blinding. Flow and timing was generally well reported. In terms of applicability, most studies were judged to have low concern, though issues were noted in a subset related to unclear test procedures or control definitions. These findings highlight the need for more rigorous and standardized methodologies in future biomarker studies to improve reliability and clinical relevance.

### Peripheral blood biomarkers

#### Peripheral eosinophil counts

As was found in the previous systematic review by Hines et al. (5), peripheral blood and peripheral blood products remain the most commonly investigated sources for non-invasive biomarkers.

In the current review, we found that peripheral blood eosinophil count (PEC) was the most commonly investigated biomarker, being investigated in 12 of the 37 studies published since July 2017 (32%). However, out of these 12 studies, only seven studies (58%) found PEC to be a significant biomarker for EoE diagnosis or disease outcomes, indicating limited reliability of PEC in EoE management. Studies that investigated PEC and/or peripheral eosinophil progenitor count (EoP) are summarized in Table 1.

**Table 1:** Studies investigating peripheral eosinophil count and/or eosinophil progenitor count as biomarkers for EoE.

References	Age group	Biomarker/s studied	Biomarker/s significant vs. non-EoE controls	Biomarker/s significant vs. atopic controls	Biomarker/s significant vs. disease activity controls
Botan et al. (9)	Pediatrics and adults	PEC	PEC	Not studied	Not studied
Choudhury et al. (44)	Pediatrics	PEC	Not studied	Not studied	PEC
Esteves Caldeira et al. (45)	Pediatrics and adults	PEC	Not studied	Not studied	No significant biomarkers
Henderson et al. (46)	Pediatrics	EoP	Not studied	Not studied	EoP
Josyabhatla et al. (32)	Pediatrics	PEC	PEC	Not studied	No significant biomarkers
Lim et al. (47)	Adults	PEC	PEC	Not studied	No significant biomarkers
Lingblom et al. (12)	Adults	PEC	Not studied	Not studied	PEC
Lu et al. (48)	Pediatrics	PEC	PEC	Not studied	Not studied
Muftah et al. (49)	Adults	PEC	Not studied	Not studied	PEC
Perez-Lucendo et al. (10)	Adults	PEC	No significant biomarkers	No significant biomarkers	No significant biomarkers
Schwartz et al. (50)	Pediatrics	PEC & EoP	Not studied	Not studied	EoP
Wechsler et al. (13)	Pediatrics	PEC	PEC	Not studied	PEC

PEC, peripheral eosinophil count; EoP, eosinophil progenitor count.

#### Peripheral eosinophil phenotype

In addition to PEC and EoP, a variety of other blood-based biomarkers were investigated in the included studies. An overview of the studies that investigated other peripheral blood biomarkers is presented in Table 2. Five studies (13.5%) investigated whether blood eosinophil phenotype could be used as a biomarker for EoE. These studies utilized flow cytometry to assess the expression levels of various surface and intracellular markers on peripheral eosinophils. Over 40 different markers were assessed across these studies.

**Table 2:** Studies investigating other blood-based biomarkers for EoE.

References	Age group	Biomarker/s studied	Biomarker/s significant vs. non-EoE controls	Biomarker/s significant vs. atopic controls	Biomarker/s significant vs. disease activity controls
Adel-Patient et al. (17)	Pediatrics	IgE, IgG1, IgG2, IgG4, IgG4, IgA, IgM, T cell subsets, ILC subsets, 80 cytokines, untargeted metabolomics	IgE, IgG1, IL17A, LIGHT, CXCL9, CXCL12, CCL15, CCL19, pyridoxine, pyridoxic acid, N-acetyl-DL-tryptophan, methionine, threonine, thymine	Not studied	Not studied
Botan et al. (9)	Pediatrics and adults	Activated eosinophil morphology, COX-2, 5-LOX	Activated eosinophil morphology, COX-2	Not studied	Not studied
Cengiz (15)	Adults	ECP	ECP	Not studied	Not studied
Esteves Caldeira et al. (45)	Pediatrics and adults	IgE	Not studied	Not studied	No significant biomarkers
Johansson et al. (11)	Adults	Surface markers on eosinophils: N29, CD62P, CD41 and 13 other markers	Not studied	Not studied	CD41 on eosinophils *(response to PPI therapy)*
Lim et al. (47)	Adults	IgE, IgG4, food-specific-IgE, food-specific-IgG4	Food-specific-IgE to wheat, eggs, food-specific-IgG4 to milk, wheat, soy, eggs, nuts	Not studied	No significant biomarkers
Lingblom et al. (12)	Adults	Markers on eosinophils/neutrophils/T cells: CD3, CD4, CD8, CD16, CD25, CD44, CD49d, CD54, CD66c, CCR3, CCR9, CD274, CD294	Not studied	Not studied	Model of 13 immune parameters (eosinophil count, lymphocyte count, T cell count, CD66c, CD294, CD44, CD25, CD193, CD16, CD49d) and 10 questionnaire scores (dysphagia, dryness, reflux, taste, milk, pasta, meat, fruit, cough, local pain) (*corticosteroid treatment responsiveness*)
Lu et al. (48)	Pediatrics	IL-1, IL-2, IL-4, IL-5, IL-6, IL-8, IL-10, IL-12, IL-13, IFN-*γ*, TNF-α, 15(S)-HETE	IL-5, IL-10, 15(S)-HETE	Not studied	Not studied
Moye et al. (51)	Pediatrics	48 plasma metabolites	dimethyl arginine, N-acetylputrescin, 2-hydroxypalmitic acid, N-acetylornithine, isoleucine, asparagine, 3hydroxyanthranilicacid, methylglutaryl carnitine	Not studied	No significant biomarkers
Pehrsson et al. (52)	Adults	PRO-C3, PC3X, C3M, CTX-III, PRO-C4, C4M, PRO-C5, PRO-C6, C6M, VICM, VIM, CPa9-HNE	PRO-C3, C3M, C4M, PRO-C5, PRO-C6, C6M, VIM, and CPa9-HNE	Not studied	No significant biomarkers
Perez-Lucendo et al. (10)	Adults	Markers on eosinophils: CD69, IL5Ra, CD44, ICAM-1, CD63	ICAM-1 on eosinophils	ICAM-1 on eosinophils	ICAM-1 on eosinophils *(inactive vs active EoE)*
Sarbinowska et al. (14)	Adults	IL-5, IL-13, TGF-*β*1, MBP, eotaxin-3	MBP and TGF-β1	Not studied	MBP, eotaxin-3 (correlated with peak esophageal eosinophils), IL-13 and TGF-β1 *(correlated with EREFS after PPI treatment)*
Sninsky et al. (53)	Adults	mRNA and miRNA sequencing	*IL5RA, RPL7AP45, GALNTL6*	Not studied	No significant biomarkers
Ugalde-Triviño et al. (20)	Adults	Immune cell counts and surface markers (39 markers)	pDC count	Not studied	pDCs and classical monocytes *(response to PPI therapy)*
Upparahalli Venkateshaiah et al. (54)	Pediatrics anD Adults	mRNA: *FceRI, FceRII, IL-15Ra, Va24, Ja18, αTCR, βTCR, γTCR, dTCR*	*Va24, Ja18, FceRI, αTCR, βTCR, γTCR, dTCR*	Not studied	Not studied
Upparahalli Venkateshaiah et al. (55)	Pediatrics and adults	mRNA: *CD101, CD274, CXCR6, VB11, CD1d, CXCL16*	*CD101, CD274, CXCR6*	Not studied	Not studied
Votto et al. (16)	Pediatrics	ECP, tryptase, GAL-10, IL-1, IL-2, IL-4, IL-5, IL-6, IL-10, IL-17, TNF-α, TNF-β, TGF-β, VEGF-A, VCAM-1, Ang-2, PAI-1, IgG4	GAL-10, TGF-β	Not studied	IL-17 *(disease activity clinical and endoscopic)*
Wechsler et al. (13)	Pediatrics	EDN, ECP, MBP-1, CLC, eotaxin-2, eotaxin-3, urine OPN and MMP-9	ECP, EDN, MBP-1, CLC, eotaxin-3, urine OPN	Not studied	ECP, EDN, MBP-1, CLC *(treatment response)*

CLC, Charcot-Leyden crystal; ECP, eosinophil cationic protein; EDN, eosinophil-derived neurotoxin; MBP, major basic protein; MMP-9, matrix metalloproteinase-9; OPN, osteopontin.

Botan and colleagues reported that an activated eosinophil morphology and intracellular expression of COX-2 in peripheral eosinophils could distinguish EoE patients from non-EoE controls (9). Perez-Lucendo et al. reported that ICAM-1 expression on blood eosinophils was reduced in active EoE patients compared to inactive EoE patients and non-EoE controls (10). Johansson et al. reported that CD41 expression on eosinophils, which is a marker for platelet adhesion to eosinophils, was predictive of responsiveness to PPI therapy (11). Finally, Lingblom et al. proposed a predictive model for distinguishing topical corticosteroid responders from non-responders, their model included the flow cytometry-detected markers CD25, CD49d, CD44, CD66c, CD193, and CD294 on eosinophils (12). Overall, phenotypic analysis of eosinophils shows some promise as biomarkers for EoE, however, consensus on which specific phenotypic marker or markers offer the greatest diagnostic and prognostic utility, is currently lacking.

#### Non-cellular circulating biomarkers

In addition to PEC and phenotypic analysis of peripheral eosinophils, other blood-based biomarkers that have been investigated for EoE including cytokines, eosinophil-derived degranulation proteins, immunoglobulins, metabolites, and circulating levels of RNA. In allergen-driven inflammatory conditions, such as EoE, eosinophils often become activated and degranulate to release degranulation proteins and cytokines, which can be detected in circulation. As a result, eosinophil-derived proteins and type 2-associated cytokines are attractive biomarkers for EoE.

Out of the 37 included studies, four studies (11%) investigated circulating levels of eosinophil degranulation proteins as potential biomarkers for EoE. The most commonly assessed were major basic protein 1 (MBP-1) and eosinophil cationic protein (ECP), which were investigated in two and three studies, respectively. Both of the studies investigating MBP-1 found that circulating levels of MBP-1 were elevated in patients with EoE compared to non-EoE controls (13, 14). Two of the studies investigating ECP similarly found that circulating levels of ECP were elevated in EoE compared to non-EoE controls (13, 15), however a third study found no difference (16).

The most commonly assessed cytokines were the type 2-associated cytokines; IL-4, IL-5, IL-13, eotaxin-2, eotaxin-3, and TGF-*β*. Out of these cytokines the most promising findings were for TGF-β and eotaxin-3. Both of the studies that investigated TGF-β found that circulating TGF-β levels were significantly higher in EoE patients compared to non-EoE controls (14, 16). Circulating eotaxin-3 was shown to be significantly elevated in EoE patients compared to non-EoE controls (13) and eotaxin-3 significantly correlated with peak esophageal eosinophil count in EoE patients (14). However, contrary to these findings, Adel-Patient et al. demonstrated no difference in eotaxin-3 levels between EoE patients and non-EoE controls (17). Overall, the non-cellular blood-based biomarkers found to be significant biomarkers for EoE in more than a single study were the eosinophil degranulation proteins MBP-1 and ECP, and the cytokines eotaxin-3 and TGF-*β*.

#### Peripheral non-eosinophil immune cells

In EoE research it is increasingly being recognized that non-eosinophil immune cells also play a critical role in driving the pathogenesis of EoE (18, 19). This understanding of the complex immunological mechanisms in EoE is reflected in studies that investigated other immune cell counts as potential complementary biomarkers. Lingblom and colleagues demonstrated that a panel comprised of 13 immune parameters (including peripheral eosinophil and T cell counts) and 10 patient-reported outcomes, was able to separate corticosteroid responsive EoE patients from non-responders (12). Ugalde-Triviño and colleagues conducted high dimensional flow cytometry analysis to evaluate the levels of different immune cell subsets in peripheral blood mononuclear cells. They showed that circulating plasmacytoid dendritic cells (pDCs) were significantly decreased in EoE patients compared to non-EoE controls. Furthermore, they found that circulating pDCs and classical monocyte counts were associated with responses to PPI therapy (20). However, another study showed that, while soluble immune constituents in circulation differed between children with EoE and controls, they did not identify any peripheral cellular constituents that differed between EoE patients and controls (17).

### Urine

Hines et al. identified two studies published prior to 2017 that investigated urine as a source of non-invasive biomarkers for EoE. Since 2017, only one study has been published that investigated urine-derived biomarkers for EoE. This study, conducted by Wechsler et al., collected urine from EoE patients and non-EoE controls and analyzed the concentration of osteopontin (OPN) and matrix metalloproteinase-9 (MMP-9). They found that urine OPN was significantly elevated in EoE patients compared to non-EoE controls (13). This result suggests that urine may represent an under-investigated source for biomarkers for EoE.

### Oral cavity and saliva biomarkers

The oral cavity is an attractive source for biomarkers for EoE due to its close physical proximity to the upper GI tract and ease of access. We identified two studies that investigated biomarkers obtained from oral cavity swabs (5% of total studies) and four studies that investigated saliva-derived biomarkers (11% of total studies). Most of these studies investigated the use of RNA molecules as biomarkers. Two studies assessed salivary microRNA (miRNA) levels. Bhardwaj et al. demonstrated that miR-4668-5p expression was elevated in EoE patients compared to controls and was reduced following corticosteroid treatment (21). Jhaveri et al. found that seven miRNAs were differentially expressed between EoE patients and controls (22). In addition, two groups conducted 16S rRNA sequencing to characterize the salivary microbiome in EoE, in order to identify potential microbiome-associated biomarkers for EoE (23, 24). Overall, while a few studies did identify one or more potential biomarker, no biomarker was identified in more than a single study, highlighting the need for further research to establish consensus on the most reliable oral cavity biomarker. Studies that investigated biomarkers for EoE derived from the oral cavity are summarized in Table 3.

**Table 3:** Studies investigating oral and exhaled biomarkers for EoE.

References	Age group	Biomarker collection/detection method	Biomarker/s studied	Biomarkers significant vs non-EoE controls	Biomarkers significant vs atopic controls	Biomarkers significant vs disease activity controls
Avinashi et al. (56)	Pediatrics	Oral cavity swab	IL-5, IL-8, IL-13, MBP, EDN, EPX	No significant biomarkers	Not studied	No significant biomarkers
Bhardwaj et al. (21)	Adults	Saliva sample	miR-4668-5p	No significant biomarkers	Not studied	miR-4668
Facchin et al. (23)	Adults	Saliva sample	16S rRNA	23 variants positively associated, and 27 variants negatively associated, with EoE	Not studied	Not studied
Hiremath et al. (24)	Pediatrics	Saliva sample	16S rRNA	*Leptotrichiaceae, Actinomyces, Lactobacillus, Strep-tococcus*	Not studied	*Haemophilus*
Jhaveri et al. (22)	Pediatrics	Buccal swab	miRNA sequencing	miR-205-5p, miR-30a-5p, miR-26b-5p, miR-27b-3p, Let-7i-5p, miR-142-5p, miR-30a-5p	Not studied	Not studied
Johnson et al. (33)	Adults	Exhaled breath	FENO	Not studied	Not studied	No significant biomarkers
Josyabhatla et al. (32)	Pediatrics	Exhaled breath	FENO	FENO	Not studied	FENO
Kaur et al. (31)	Pediatrics and adults	Exhaled breath	FENO	FENO	Not studied	Not studied
Sebastian-Delacruz et al. (57)	Adults	Oral cavity swab	Expression of 29 genes	*CDH26, KCNJ2, PLD1*	Not studied	No significant biomarkers

EDN, eosinophil-derived neurotoxin; EPX, eosinophil peroxidase; FENO, fractional exhaled nitric oxide; MBP, major basic protein.

### Exhaled breath

Human breath is a useful source of biological information and has various benefits including being non-invasive, well-tolerated, easy to collect, and amenable to repeat sampling (25). One of the most studied breath-based markers is fractional exhaled nitric oxide (FeNO), a standardized clinical tool for assessing eosinophilic airway inflammation (26, 27) FeNO is well established in asthma and has been shown to correlate with eosinophilia and airway inflammation (28–30).

Since 2017, three studies have evaluated FeNO in EoE. Kaur et al. (31) found that FeNO levels were significantly higher in patients with EoE compared to non-EoE controls. Josyabhatla et al. (32) similarly reported elevated FeNO in active EoE compared to both inactive EoE and non-EoE controls, suggesting that FeNO may have utility in distinguishing disease activity. They also proposed that FeNO, when combined with other blood-based biomarkers, could enhance diagnostic accuracy, although no formal combination model was tested. In contrast, Johnson et al. (33) assessed FeNO as a standalone diagnostic tool and found that a cutoff value >40 ppb had high specificity (0.94) but poor sensitivity (0.16) for detecting histologic activity (>15 eos/hpf). This high false-negative rate led the authors to conclude that FeNO alone is insufficient for diagnosing or monitoring EoE.

Taken together, these findings indicate that while FeNO may have limited diagnostic utility on its own, it could contribute to a multi-marker panel for EoE assessment. However, further studies are needed to evaluate combinations of FeNO with other promising biomarkers—such as eosinophil-derived neurotoxin (EDN), periostin, or cytokines like eotaxin-3—to determine whether such combinations improve accuracy for diagnosis or monitoring. Studies that investigated biomarkers for EoE derived from the oral cavity, saliva or exhaled breath are summarized in Table 3.

### Biomarkers from esophageal contents or esophageal physiological testing

In the previous 2019 review, Hines et al. identified three studies that used either the Cytosponge or the esophageal string test (EST) for minimally-invasive esophageal sampling (5). Two studies from the same group utilized the Cytosponge, focusing on eosinophil counts and eosinophil-derived neurotoxin (EDN) as biomarkers to assess disease activity and distinguish active EoE from remission (34, 35). One study employed the EST in a pediatric population, analyzing eosinophil-associated proteins such as MBP, EDN, ECP, and Charcot-Leyden crystal protein (CLC) (36).

Since 2017, three further studies have explored Cytosponge and EST as collection methods for EoE biomarkers and, in addition, two studies have explored esophageal brushing samples, and one study explored multichannel pH impedance. Table 4 summarizes the studies that investigated biomarkers from esophageal contents or esophageal physiological testing.

**Table 4:** Studies investigating biomarkers from esophageal contents or esophageal physiological testing.

References	Age group	Biomarker collection/detection method	Biomarker/s Studied	Biomarker/s significant vs. non-EoE controls	Biomarker/s significant vs. atopic controls	Biomarker/s significant vs. disease activity controls
Ackerman et al. (37, 38)	Pediatrics and adults	EST	EDN, EPX, MBP-1, CLC, Eotaxin-2, Eotaxin-3	EDN, EPX, MBP-1, CLC, eotaxin-2, eotaxin-3	Not studied	Eotaxin-3 and MBP-1 *(active vs. inactive EoE)*
Eldredge et al. (42)	Pediatrics	Multichannel pH impedance	Baseline impedance	Baseline impedance	Not studied	Not studied
Katzka et al. (37, 41)	Adults	Cytosponge	Esophageal eosinophil count	Esophageal eosinophil count	Not studied	Esophageal eosinophil count *(active vs. inactive EoE)*
Muir et al. (39, 40)	Pediatrics	EST	EDN, EPX, MBP-1, CLC, Eotaxin-3, Periostin	EDN, EPX, MBP-1, CLC, Eotaxin-3, Periostin	Not studied	Not studied
Smadi et al. (38, 40)	Pediatrics and adults	Esophageal brushing via endoscopy or nasogastric tube	EDN	EDN	Not studied	EDN *(active vs. inactive EoE)*
Thomas et al. (39, 41)	Pediatrics and adults	Endoscopic esophageal brushing	EDN	EDN	Not studied	EDN *(active vs. inactive EoE, EREFS)*

CLC, Charcot-Leyden crystal; EDN, eosinophil-derived neurotoxin; EPX, eosinophil peroxidase; EST, esophageal string test; MBP, major basic protein.

### Cytosponge

The Cytosponge has shown promise as a minimally invasive tool for EoE monitoring. Originally designed for esophageal cancer screening, the Cytosponge consists of a capsule containing a compressed mesh sponge that expands once swallowed and collects cellular material as it is withdrawn through the esophagus (34). We identified one study published since 2017 that utilized the Cytosponge, which demonstrated high correlation between Cytosponge-based and biopsy-based eosinophil counts, showing a sensitivity of 75% and specificity of 86% for detecting active EoE (37).

### Esophageal string test

The EST involves a swallowed nylon string that captures eosinophil-associated proteins from esophageal secretions (36). We found two studies that used this collection tool, from the same collaborative groups (38, 39). In both studies, the EST effectively captured eosinophil-associated proteins, including eotaxin-3, MBP-1, EDN, eosinophil peroxidase (EPX), and CLC. These biomarkers showed significant correlations with peak eosinophil counts, endoscopic scores, and markers of epithelial-mesenchymal transition. Using the EST Ackerman et al. found that eotaxin-3 in the esophagus had the greatest sensitivity and specificity for predicting active vs. inactive disease (38). In addition, patient preference strongly favored the EST over endoscopy, with 87% of children, 95% of parents, and 92% of adults preferring the minimally invasive method if it provided equivalent diagnostic information.

### Esophageal brushings

We identified two studies from the same group that investigated esophageal brushings as a non-invasive method for the collection of biomarker samples. Esophageal brushing was performed either through endoscopy (40, 41) or via a nasogastric tube (NGT) (40).

In the first study, EDN concentrations in brushing samples were significantly higher in patients with active EoE compared to those with GERD, EoE in remission, or no disease. An EDN threshold of ≥10 mcg/mL reliably identified active EoE with a sensitivity and specificity of 97 and 89%, respectively. The NGT brushing method was well-tolerated, safe, and provided a less invasive alternative to endoscopic brushing for detecting and monitoring EoE inflammation (40). In the second study, EDN concentrations were significantly higher in patients with active EoE compared to those in remission or controls, and EDN levels correlated strongly with the EoE Endoscopic Reference Score (EREFS) and peak eosinophil count (41).

### Mucosal impedance

In a single retrospective study children with EoE underwent multichannel pH impedance (pH-MII) testing. The children with EoE demonstrated significantly lower baseline impedance across all esophageal segments (upper, mid, and lower), compared to controls, consistent with poor mucosal integrity in EoE. However, the study did not find a direct correlation between baseline impedance values and eosinophil counts at corresponding esophageal segments (42).

## Discussion

EoE remains a challenging condition to diagnose and monitor, primarily due to the reliance on invasive procedures such as endoscopy and biopsy. This systematic review summarizes the progress in the development of non-invasive biomarkers for EoE, highlighting future potential for new ways of diagnosing and monitoring the disease.

### Peripheral blood biomarkers

Peripheral eosinophil count is the most commonly studied biomarker. Blood-based biomarkers are popular in clinical settings due to the ease of sampling, the potential range of systemic biomarkers, and the ability for repeat measures to be taken. However, for EoE, PEC demonstrated statistical significance in only 58% of studies for EoE diagnosis or disease outcomes. This limited reliability highlights the need for alternative biomarkers. Emerging approaches, such as evaluating eosinophil phenotype and combinatorial immune cell signature via multi-parameter flow cytometry, show promise. With the rapid advancement of multi-parameter flow cytometry, this is becoming a powerful and popular methodology for the detection of immune biomarkers (43). These techniques can identify specific phenotypic states of circulating immune cells, which may better correlate with EoE disease activity. Circulating soluble biomarkers, including eosinophil degranulation products (e.g., MBP-1, ECP) and cytokines (e.g., eotaxin-3, TGF-*β*), also hold potential.

While blood-based biomarkers have many advantages for disease diagnosis and monitoring, one of the main concerns around the use of blood-based biomarkers is that systemic biomarkers may lack specificity and sensitivity. Larger studies will be required to establish their specificity and sensitivity for EoE and their ability to distinguish EoE from other type 2 diseases, such as asthma, atopic dermatitis and allergic rhinitis. Of note, out of the 37 studies included in this review only the study by Perez-Lucendo and colleagues (10) included a separate control group with atopy. While blood-based biomarkers remain valuable, more research needs to be conducted to confirm their specificity for EoE, in particular in comparison to other allergic conditions.

### Oral cavity biomarkers

Non-invasive biomarker sources like oral cavity swabs saliva and exhaled breath offer attractive alternatives. To date, studies investigating oral cavity swabs and saliva have yielded mixed results. A number of studies have looked at RNA molecules, which hold potential as sensitive and specific biomarkers. Further investigation is warranted into the potential application of these molecular biomarkers. Similarly, FENO in exhaled breath has produced mixed results and overall has limited specificity for EoE. These methods will require refinement or combination with other biomarkers to improve diagnostic accuracy.

### Esophageal-derived biomarkers

Direct sampling from the esophagus remains the most promising approach, with non-invasive methods like the Cytosponge, esophageal string test (EST), and esophageal brushing showing significant utility. These tools reliably measure the eosinophil-derived protein, EDN, and other inflammatory markers, which have been shown to correlate strongly with histologic eosinophil counts and disease activity. Importantly, these methods are better tolerated and more cost-effective than endoscopy, making them viable options for routine monitoring.

### Pediatric vs. adult populations

Of the studies reviewed, 14 were conducted in pediatric populations, 15 in adult populations, and eight included both pediatrics and adults. Differences in EoE pathogenesis and presentation between adults and children necessitate consideration of differences in biomarker discovery and potential utility between the age groups. Pediatric EoE is often characterized by symptoms such as feeding difficulties and failure to thrive, while adults frequently present with dysphagia and food impaction. These differences may influence biomarker expression and utility, and future studies must carefully stratify and analyze these populations to ensure the generalizability of findings across age groups. Non-invasive biomarkers are particularly crucial for pediatric patients, as the burden of repeated endoscopy is greater in this vulnerable population.

### Future directions

The future of non-invasive biomarker development lies in head-to-head comparisons of emerging biomarkers and collection methods against a defined gold standard, such as eosinophil counts from biopsies. Studies must evaluate the specificity of identified biomarkers across various eosinophilic and atopic conditions to ensure they are able to reliably differentiate EoE. Incorporating minimally invasive tools into clinical practice is imperative, especially for monitoring disease activity and treatment response in children. A key limitation in biomarker discovery for EoE is the considerable heterogeneity among investigated biomarkers, including the use of different techniques and threshold criteria across studies. In addition, it is important to consider that a combination of multiple bioamrkers may provide the most effective strategy for EoE disease monitoring and management, and this should be investigated in future studies. In conclusion, while a wide variety of biomarkers have been evaluated for EoE and significant progress has been made, the field requires robust, comparative studies to establish a consensus of the best methods and biomarkers, or combination of biomarkers, for routine use. Non-invasive biomarkers demonstrate significant potential to reduce the burden of invasive procedures and improve the quality of care for patients with EoE.

## Data Availability

The original contributions presented in the study are included in the article/Supplementary material, further inquiries can be directed to the corresponding author.
