# Supplementary material for: Non-invasive biomarkers to diagnose and monitor eosinophilic esophagitis: a systematic review
Source: Front Med (Lausanne). 2025 Jun 26;12:1607306. doi: 10.3389/fmed.2025.1607306 (PMC12240998; doi:10.3389/fmed.2025.1607306)
Supplement: Supplementary file 1 [file Table_1.docx]

**Supplementary Material 1**

# Search Strategy Concepts and Keywords

|  | **Concept 1: Population** | **Concept 2: Intervention** | **Concept 3: Comparator** |
| --- | --- | --- | --- |
| **Key concepts** | Eosinophilic esophagitis patients | Use of non-invasive biomarker | Standard invasive methods or no biomarkers |
| **Free text terms / natural language terms** | (esophag* OR oesophag*) AND (eosinophil* OR hypereosinophil*) | (marker* OR biomarker* OR bio-marker* OR serologic* marker*) AND (noninvasive OR non-invasive OR minimally invasive OR semi-invasive OR semi invasive OR non-endoscop* OR nonendoscop* OR cytosponge OR sponge OR brush OR string OR capsule) |  |

# PubMed Search Strategy

| **PubMed**  **Filters:**  July 1^st^ 2017 to July 19^th^ 2024 | #1 | (((("esophag*"[Title/Abstract] OR "oesophag*"[Title/Abstract]) AND ("eosinophil*"[Title/Abstract] OR "hypereosinophil*"[Title/Abstract]) AND ("marker*"[Title/Abstract] OR "biomarker*"[Title/Abstract] OR "bio marker*"[Title/Abstract] OR "serologic* marker*"[Title/Abstract]) AND ("noninvasive"[Title/Abstract] OR "non-invasive"[Title/Abstract] OR "minimally invasive"[Title/Abstract] OR "semi-invasive"[Title/Abstract] OR "non endoscop*"[Title/Abstract] OR "nonendoscop*"[Title/Abstract] OR "cytosponge"[Title/Abstract] OR "sponge"[Title/Abstract] OR "brush"[Title/Abstract] OR "string"[Title/Abstract] OR "capsule"[Title/Abstract])) NOT "Review"[Publication Type]) AND 2017/07/01:2024/07/19[Date - Publication]) AND (2017/7/1:2024/7/19[pdat]) |
| --- | --- | --- |

# Web of Science Search Strategy

| **Web of Science**  **Filters:**  July 1^st^ 2017 to July 19^th^ 2024 | #1 | Topic:  ((TS=(esophag*)) OR TS=(oesophag*)) AND ((TS=(eosinophil*)) OR TS=(hypereosinophil*)) AND ((TS=(marker*)) OR TS=(biomarker*) OR TS=(bio-marker*) OR TS=(serologic* marker*)) AND ((TS=(noninvasive)) OR TS=(non-invasive) OR TS=(minimally invasive) OR TS=(semi-invasive) OR TS=(semi invasive) OR TS=(non-endoscop*) OR TS=(nonendoscop*) OR TS=(cytosponge) OR TS=(sponge) OR TS=(brush) OR TS=(string) OR TS=(capsule)) NOT DT=(Review) |
| --- | --- | --- |

# Embase/Medline Search Strategy

| **Embase**  **Filters:**  July 1^st^ 2017 to July 19^th^ 2024 | #1  #2  #3  #4  #5 | esophag* OR oesophag*  AND  eosinophil* OR hypereosinophil*  AND  marker* OR biomarker* OR bio-marker* OR serologic* marker*  AND  noninvasive OR non-invasive OR minimally invasive OR semi-invasive OR semi invasive OR non-endoscop* OR nonendoscop* OR cytosponge OR sponge OR brush OR string OR capsule  NOT  Review |
| --- | --- | --- |
