# Supplementary material for: Non-invasive biomarkers to diagnose and monitor eosinophilic esophagitis: a systematic review
Source: Front Med (Lausanne). 2025 Jun 26;12:1607306. doi: 10.3389/fmed.2025.1607306 (PMC12240998; doi:10.3389/fmed.2025.1607306)
Supplement: Supplementary file 2 [file Table_2.docx]

| **Author** | **Year** | **Test** | **Risk of bias** | | | |  | **Applicability concerns** | | | |
| --- | --- | --- | --- | --- | --- | --- | --- | --- | --- | --- | --- |
|  |  |  | **P** | **I** | **R** | **FT** |  | **P** | **I** | **R** | **FT** |
| Katzka *et al* | 2017 |  | ✓ | ✓ | ✓ | ✓ |  | ✓ | ✓ | ✓ | ✓ |
| Botan *et al* | 2017 |  | ✓ | ✗ | ✓ | ✓ |  | ✓ | ✗ | ✓ | ✓ |
| Lu *et al* | 2018 |  | ✗ | ✓ | ✓ | ✓ |  | ✗ | ✓ | ✓ | ✓ |
| Smadi *et al* | 2018 |  | ✗ | ✗ | ✓ | ✓ |  | ✗ | ✗ | ✓ | ✓ |
| Moye *et al* | 2019 |  | ✗ | ✓ | ✓ | ✓ |  | ✗ | ✓ | ✓ | ✓ |
| Johnson *et al* | 2019 |  | ✓ | ✓ | ✓ | ✓ |  | ✓ | ✓ | ✓ | ✓ |
| Ackerman *et al* | 2019 |  | ✓ | ✓ | ✓ | ✓ |  | ✓ | ✓ | ✓ | ✓ |
| Cengiz | 2019 |  | ✗ | ✗ | ✓ | ✗ |  | ✗ | ✗ | ✓ | ✗ |
| Avinashi *et al*. | 2019 |  | ✓ | ✓ | ✓ | ? |  | ✗ | ✓ | ✓ | ? |
| Hiremath *et al* | 2019 |  | ✗ | ✗ | ✓ | ✓ |  | ✗ | ✗ | ✓ | ✓ |
| Schwartz *et al* | 2019 |  | ✓ | ✓ | ✓ | ✓ |  | ✓ | ✓ | ✓ | ✓ |
| Johansson *et al* | 2020 |  | ✓ | ✓ | ✓ | ✓ |  | ✓ | ✓ | ✓ | ✓ |
| Lingblom *et al* | 2020 |  | ✗ | ✓ | ✓ | ✓ |  | ✗ | ✓ | ✓ | ✓ |
| Bhardwaj *et al* | 2020 |  | ? | ✓ | ✓ | ? |  | ✗ | ✗ | ✓ | ? |
| Choudhury *et al* | 2020 |  | ✗ | ✗ | ✓ | ✓ |  | ✗ | ✗ | ✓ | ✓ |
| Henderson *et al* | 2020 |  | ✗ | ? | ✓ | ✓ |  | ✗ | ? | ✓ | ✓ |
| Sarbinowska *et al* | 2021 |  | ✓ | ✓ | ✓ | ✓ |  | ✓ | ✓ | ✓ | ✓ |
| Perez-Lucendo *et al* | 2021 |  | ? | ✓ | ✓ | ✗ |  | ? | ✓ | ✓ | ✗ |
| Venkateshaiah *et al* | 2021 |  | ✗ | ? | ? | ? |  | ✗ | ? | ? | ? |
| Venkateshaiah *et al* | 2021 |  | ✗ | ? | ✓ | ✓ |  | ✗ | ? | ✓ | ✓ |
| Wechsler *et al* | 2021 |  | ✗ | ✓ | ✓ | ✗ |  | ✗ | ✓ | ✓ | ✗ |
| Eldredge *et al* | 2022 |  | ✗ | ✗ | ✓ | ✓ |  | ✗ | ✗ | ✓ | ✓ |
| Muir *et al* | 2022 |  | ✗ | ✓ | ✓ | ✓ |  | ✗ | ✓ | ✓ | ✓ |
| Facchin *et al* | 2022 |  | ✓ | ✗ | ✓ | ✓ |  | ✓ | ✗ | ✓ | ✓ |
| Adel-Patient *et al* | 2023 |  | ✗ | ✓ | ✓ | ✓ |  | ✗ | ✓ | ✓ | ✓ |
| Caldeira *et al* | 2023 |  | ? | ✗ | ✓ | ✓ |  | ? | ✗ | ✓ | ✓ |
| Jhaveri *et al* | 2023 |  | ✗ | ✓ | ✓ | ✓ |  | ✗ | ✓ | ✓ | ✓ |
| Josyabhatla *et al* | 2023 |  | ✗ | ✓ | ✓ | ✓ |  | ✗ | ✓ | ✓ | ✓ |
| Kaur *et al* | 2023 |  | ✓ | ✓ | ✓ | ✓ |  | ✓ | ✓ | ✓ | ✓ |
| Pehrsson *et al* | 2023 |  | ✗ | ✓ | ✓ | ✓ |  | ? | ✓ | ✓ | ✓ |
| Thomas *et al* | 2023 |  | ✗ | ✓ | ✓ | ✓ |  | ✗ | ✓ | ✓ | ✓ |
| Votto *et al* | 2023 |  | ✗ | ? | ? | ? |  | ✗ | ? | ? | ? |
| Lim *et al* | 2024 |  | ✓ | ✓ | ✓ | ✓ |  | ✓ | ✓ | ✓ | ✓ |
| Muftah *et al* | 2024 |  | ✗ | ✓ | ✓ | ✓ |  | ✗ | ✓ | ✓ | ✓ |
| Maialen *et al* | 2024 |  | ✗ | ✗ | ✓ | ? |  | ✗ | ✗ | ✓ | ? |
| Sninsky *et al* | 2024 |  | ✓ | ? | ✓ | ✓ |  | ✓ | ? | ✓ | ✓ |
| Ugalde-Trivino *et al* | 2024 |  | ✓ | ? | ✓ | ✓ |  | ✓ | ? | ✓ | ✓ |

P = Patient selection; I = Index test; R = Reference standard; FT = Flow and timing.
✓ indicates low risk; ✗ indicates high risk; ? indicates unclear risk.

Table 2: QUADAS-2 Eosinophilic Esophagitis Minimally Invasive Biomarker Studies risk of bias and applicability judgments
